# Drawing soldiers out of post-traumatic stress disorder

**DOI:** 10.1186/s40779-019-0195-8

**Published:** 2019-02-18

**Authors:** Simon R. Hunter

**Affiliations:** Nelson Marlborough Institute of Technology, 322 Hardy Street, Nelson, 7010 New Zealand

**Keywords:** Adjuvant therapy, Art therapy, Drawing, Painting, Post-traumatic stress disorder

## Abstract

Art therapies are a broad suite of treatments including drawing, painting and music that can facilitate non-verbal communication through artistic expression. They have been used as adjunctive therapies for a range of mental health conditions. Significant numbers of returning military personnel experience post-traumatic stress disorder (PTSD), and discontinuation of care is a concern. Using drawing-based art therapy as an adjuvant to classical therapies may provide a benefit for such military patients.

**Dear Editors:**

Art therapies are a broad suite of treatments including drawing, painting, dance, and music (Fig. 1) that facilitate non-verbal communication through artistic expression. Particular modalities of art therapy, such as drawing, may have a role in the treatment of post-traumatic stress disorder (PTSD) [1].

**Fig. 1:**
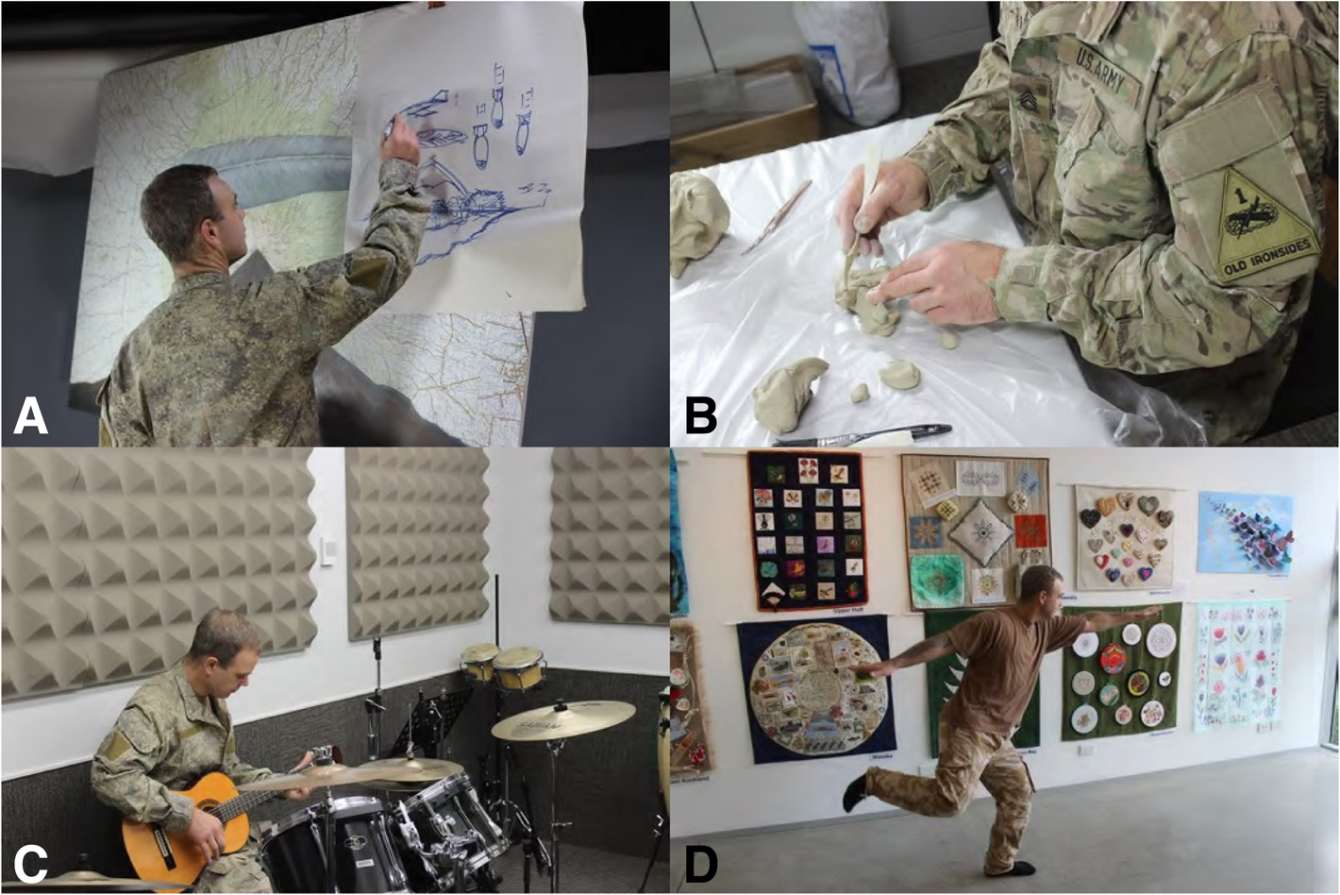
Art therapy consists of drawing and painting, manual manipulation, music or dance. **a** Drawing and painting are widely used art therapy mediums due to ease of use, portability and straightforward standardization. These art forms can facilitate a narrative of memories and feelings associated with trauma allowing the patients to distance themselves from traumatic memories. The art therapist facilitates slow and progressive detailed reliving of trauma by presenting specific questions combined with drawing and painting tasks that address major themes of the trauma one memory at a time. **b** Through manual manipulation, clay or sculpting provides an external and alternative perspective, encouraging three-dimensional thinking and the use of tactile senses. It includes the use of material that comes from the earth and often other products such as Plasticine, Play-Doh, and Fimo clay. Patients are encouraged to focus on their breathing as they manipulate the material that promotes the release of stressful emotions and memories. **c** The emotional expression music allows it to be a promising form of art therapy for those refractory to other interventions. Music therapy typically includes group sessions as well as individuals playing instruments and listening to music while being guided by a trained music therapist. **d** Dance therapy helps patients express themselves through simple rhythmic movements leading to a controlled release of traumatic memories, thoughts and experiences. Group sessions begin slowly in a circular formation with a physical warm-up. As patients become comfortable with themselves and each other, the therapist will guide them in structured movements that encourage spontaneous self-expression and interaction with others. The session closes with a period of relaxation and discussion of the experience

PTSD rates vary from 13.5% [2] to as high as 30% [3] and are influenced by the deployment location, duration of deployment and role of individual in the conflict. Symptoms of PTSD include anger, anxiety, avoidance, withdrawal, sleeplessness, increased arousal, and hypervigilance as an exaggerated form of awareness. Established treatments for PTSD include prolonged exposure (PE) therapy, cognitive processing therapy (CPT), eye movement desensitization and reprocessing (EMDR), and other psychotherapies. These interventions vary in efficacy and 24% of United States military personnel returning from Afghanistan who were diagnosed with PTSD discontinued care [4]. Discontinuation of care may potentially occur because these established interventions are not well suited for individuals drawn to military service.

Initial studies of various art therapies suggest efficacy of these nonverbal communication tools in relieving anxiety and depression, improving ability to focus, and breaking down avoidance strategies [1]. Potentially, each form of art therapy has its own strengths and can be tailored to meet individual needs depending on the primary intervention. A previous systematic review suggests that art therapy is most effective when used in combination with other conventional therapies [5]. Importantly for many patients, art therapy encourages improved social interactions and a reduction in avoidance and hypervigilance symptoms. These alternative communication methods may facilitate healing. Particularly promising relationships for exploration include the following:

1. Drawing and painting to facilitate verbalization because such activities allow expression through a fundamentally different route from speaking [6];
2. Music for depression due to the emotive nature of music and its ability to decrease anxiety in patients [7];
3. Body-oriented and movement psychotherapies for avoidance behaviours because of the group interaction, working towards a common goal, and working through traumatic memories in a kinaesthetic way [8];
4. Clay and sculpture for permissive expression of anger and cathartic release on an inanimate object and ultimately creating with the hands rather than harming [9].

The remarkable interplay between PTSD symptoms and the purported effects of drawing-based art therapy suggest that this complementary therapy may be valuable as part of a holistic treatment plan. This intriguing synergy seems appealing, as art therapy serves as an alternative means of expressing thoughts, memories and feelings for those challenged by verbal-focused therapies. While limited to case studies and small controlled trials, data involving drawing-based art therapy as a supplemental treatment for PTSD are encouraging [10]. These existing preliminary data are sufficient to support further investigation into drawing-based art therapy for PTSD through well-designed interdisciplinary trials.

The calling to serve in the military is answered by dedicated individuals who undergo specialized rigorous training to allow them to perform acts that some may consider unconscionable, however necessary. Similarly, the techniques for treating these battle-hardened military personnel potentially should not be identically transferred from the civilian sector, as these individuals are not civilians. This assertion is supported by the fact that military service personnel often experience repeated and extended trauma and feel exposure to traumatic losses. These experiences can be morally compromising.

If drawing-based art therapy is effective, it cannot be without risk. Specifically, there are case reports of art therapy potentially triggering treatment dropout [11]. Drawing and painting may show potential for alleviating PTSD symptoms because communicating and repackaging traumatic memories through this route may be less threatening than classic approaches [12]. While all interventions involve cost and risk, as drawing-based art therapy should be used as an adjuvant in a holistic treatment plan, the risk of trauma is typically already managed.

For art therapy to be more broadly implemented in the treatment of military personnel with PTSD, further research needs to be conducted. Specifically, such investigation would include comparing the current treatment practice to that with a standardized adjuvant application of art therapy.

## Conclusions

Art therapies consist of many modalities that may be particularly suited as adjuvant therapies for specific disorders. Drawing-based art therapy holds promise for the treatment of PTSD. While rigorous controlled trials are necessary, there is little reason not to consider drawing-based art therapies in combination with classic treatment approaches. Finally, some military personnel with PTSD may feel the same as the American painter and printmaker Edward Hopper: “If I could say it in words, there would be no reason to paint”.
